# Therapeutic Application of Mesenchymal Stem Cells Derived Extracellular Vesicles for Immunomodulation

**DOI:** 10.3389/fimmu.2019.02663

**Published:** 2019-11-15

**Authors:** Marina O. Gomzikova, Victoria James, Albert A. Rizvanov

**Affiliations:** ^1^Institute of Fundamental Medicine and Biology, Kazan Federal University, Kazan, Russia; ^2^M.M. Shemyakin–Yu.A. Ovchinnikov Institute of Bioorganic Chemistry of the Russian Academy of Sciences, Moscow, Russia; ^3^School of Veterinary Medicine and Science, University of Nottingham, Nottingham, United Kingdom

**Keywords:** extracellular vesicles, microvesicles, immunosuppression, autoimmune diseases, multiple sclerosis, transplant rejection, type 1 diabetes, graft-vs.-host disease

## Abstract

The immunosuppressive potential of mesenchymal stem cells has been extensively investigated in many studies *in vivo* and *in vitro*. In recent years, a variety preclinical and clinical studies have demonstrated that mesenchymal stem cells ameliorate immune-mediated disorders, including autoimmune diseases. However, to date mesenchymal stem cells have not become a widely used therapeutic agent due to safety challenges, high cost and difficulties in providing long term production. A key mechanism underpinning the immunomodulatory effect of MSCs is the production of paracrine factors including growth factors, cytokines, chemokines, and extracellular vesicles (EVs). MSCs derived EVs have become an attractive therapeutic agent for immunomodulation and treatment of immune-mediated disorders. In addition to many preclinical studies of MSCs derived EVs, their beneficial effects have been observed in patients with both acute graft-vs.-host disease and chronic kidney disease. In this review, we discuss the current findings in the field of MSCs derived EVs-based therapies in immune-mediated disorders and approaches to scale EV production for clinical use.

## Introduction

Mesenchymal stem cells (MSCs) bear great potential not only in regenerative medicine, but they also interfere with different pathways of the immune response and exhibit immunomodulatory activity (1, 2). Preclinical and clinical studies both indicate that MSCs have immunosuppressive activity, including suppression of T- and B-cell proliferation, modulation of regulatory T cell function maturation and activation, antigen presentation by dendritic cells, decrease secretion of proinflammatory cytokines and cytotoxicity (3). Up to now MSCs have been exploited in 67 clinical trials (http://clinicaltrials.gov/; accessed April 2019) of inflammation-associated diseases, autoimmune diseases and transplant rejection (Supplementary Table 1).

The accumulating evidence supports MSCs producing a strong paracrine action on neighboring cells through a broad range of growth factors, chemokines, cytokines and extracellular vesicles (EVs) (4). EVs are a heterogeneous population of spherical membrane vesicles, containing biologically active cargo of molecules (proteins, lipids, mRNA, microRNA, siRNA, miRNA, ssDNA, dsDNA) deliverable to target cells (5, 6). Due to the fundamental function of EV—mediation of intercellular communication, they are involved in numerous physiological and pathological processes and pathways (7). MSCs derived EVs have been used to recapitulate some of the biological activity of parent cells, including stimulation of regeneration in models of renal, heart, liver and nervous tissue injury (8–12) a function which is arguable the most prominent function of MSCs.

Due to the genetic instability (13), undesired differentiation (14, 15), and pulmonary embolism risks (16), the application of MSCs in a clinical setting is restricted. Cell-free therapy based on EVs offers a promising alternative to stem cell-based therapy for inflammation-associated diseases, autoimmune diseases, and transplant rejection (17–19).

A caveat in pursuing EVs for clinical therapeutic applications has been the difficulty of producing sufficient yield. Therefore, in parallel with investigation of biological activity and therapeutic efficacy, approaches to scale EV production are also underway (17, 20, 21). Large scale EV production research aims to generate an industrially feasible approach that retains clinical-grade purity. As well as alternative approaches to isolate other EV-like and larger particles (21–23).

MSCs derived EVs are a promising therapeutic instrument which have advantages over cell therapy. In this review, we focus on the recent findings of therapeutic application of MSCs derived EVs for the treatment of immune-mediated disorders and the perspective application of different methods for large scale EVs production.

## MSCs-based Immunosuppressive Therapy

*In vitro* and *in vivo* studies have been used to demonstrate the MSCs anti-inflammatory and immunomodulatory properties on both innate (macrophages, NK cells, dendritic cells) and adaptive immune cells (T-cells, B-cells) (24).

Using blocking antibodies and inhibitors it was shown that indoleamine 2,3-dioxygenase (IDO) (25), prostaglandin E2 (PGE2) (26), interleukin 10 (IL-10) (27), nitric oxide (NO) (28), and hepatocyte growth factor (HGF) (29) mediate the inhibitory action of MSCs on immune cells. The list of soluble factors which are associated with the immunomodulation capacity of MSCs is still to be fully elucidated.

The first clear evidence of MSCs immunosuppression of immune cells was produced *in vitro* by Aggarwal and Pittenger (30) by demonstrating human MSCs altered the cytokine secretion profile of co-cultured dendritic cells (DCs), T cells (TH1 and TH2), and natural killer (NK) cells to induce a more anti-inflammatory phenotype. In addition, the number of regulatory T cells (T-regs) was also increased (30). Successful immunosuppression of immune cells by MSCs *in vitro* lead to increasing the number of *in vivo* research and preclinical trials.

In more recent studies, MSCs isolated from human umbilical have been shown to inhibit inflammation in a rodent model of acute allergic rhinitis (31). Reduced expression of interleukin 4, tumor necrosis factor alpha (TNF-α), and immunoglobulin E were detected in the serum of animals treated with MSCs (31).

Similar findings have also been reported in rodent models of diabetic nephropathy (DN) and rheumatoid arthritis (RA), within which treatment with MSCs increased the concentration of anti-inflammatory cytokines (IL-10 in DN and RA and EGF in DN) This change was accompanied by a decrease in pro-inflammatory cytokines [IL-6, MCP-1, TNF-α and IL-1β in DN and of IL-6, TNF-α, TGF-β, NF-κB, toll-like receptor-2, MMP-3, COMP-1, and RF (rheumatoid factor) in RA] (32, 33).

The effects of MSCs has also been tested in autoimmune encephalomyelitis (EAE) (the animal model of multiple sclerosis). Treatment with embryonic stem cell-derived MSCs in the cynomolgus monkey EAE model reduced the clinical symptoms of brain lesions and neuronal demyelination (34).

The first clinical trial using bone marrow derived MSCs was conducted in 2006 at the University of Cambridge (Supplementary Table 1). As a result of the trial the authors suggested improvements for the design of future studies to increase the efficacy of evaluation (35).

These recommendations have led to a growth in clinical trials in the area of MSCs based treatment of autoimmune disorders (Supplementary Table 1). China is currently leading on clinical trials within this field (26.87% of total number of trials), with a predominance (44.4%) toward the treatment of type 1 Diabetes (Supplementary Table 1). Trials conducted in other countries are reviewing MSCs effects in multiple sclerosis (35.82%), type 1 diabetes (22.39%), and rheumatoid arthritis (17.91%) (Supplementary Table 1).

Whilst the comparison of the data produced in these trials is complicated by variations in design (MSCs administration, dose and regimens). Importantly, to date there have been no reports of tumor development following MSCs infusion (36, 37).

Despite promising clinical studies, MSCs have not become a universal therapeutic agent for the treatment of immune-mediated disorders (38). In the main, this is due to concerns over safety (transformation, undesired differentiation and blood vessels occlusion), and the labor intensive, industrially inapplicable procedure of preparation. It is known that the beneficial effects of MSCs are largely mediated by paracrine factors. Cell-free therapy based on mediators of cell-cell communication, of which EVs are considered a promising approach, are now considered to be a safer alternative to entire stem cell therapy (17). MSCs derived EVs offer compelling advantages over cell therapies in terms of improved biodistribution, lower toxicity, higher stability in the circulation and scalable production (39). Autologous and allogeneic MSCs can be used for the production of EVs-based therapeutics. The biocompatibility of autologous MSCs offers an attractive therapeutic approach. However, from a manufacturing perspective EVs derived from allogeneic MSCs can be obtained in higher concentrations and isolated from MSCs obtained from younger donors which are more biologically active and potentially more readily available (40).

## Mesenchymal Stem Cells Derived Extracellular Vesicles

Originally it was thought the beneficial effects of MSCs based therapy in tissue regeneration was due to the engraftment and differentiation of MSCs within damaged tissues. Subsequently, it was found that relatively few transplanted MSCs engraft in host tissues (41, 42). Together with evidence that media conditioned by MSCs is sufficient to stimulate regeneration (43, 44), the development of the paracrine hypothesis of the therapeutic effects of MSCs was formed (45), the hypothesis that is now widely accepted. Within the broad spectrum of factors secreted by MSCs, EVs have been highlighted as a potential therapeutic alternative to MSCs use (46).

**Figure 1:**
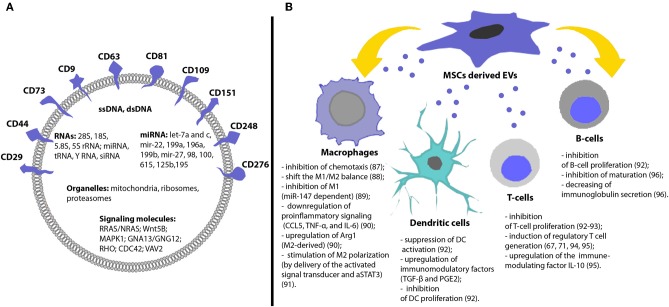
Molecular content and immunomodulatory effects of MSCs derived EVs. **(A)** EVs have specific membrane markers and contain various proteins, lipids, nucleic acids, and organelles. **(B)** MSCs derived EVs can induce different immunosuppressive effects and contribute to the immunological tolerance [for detailed information, please refer to (47–58)].

EVs are bilipid membrane vesicles, encapsulating proteins (including transcription factors, growth factors, and enzymes) and genetic material (mRNA, siRNA, miRNA, ssDNA, dsDNA) (Figure 1A) (5, 5, 6, 59). It was shown that most RNAs (>80%) inside of MSCs derived EVs are 28S, 18S, 5.8S, 5S ribosomal RNA, alongside other small RNAs miRNA (44%), tRNA (47%) and Y RNA (8%). MSCs derived EVs were enriched with miRNA which regulate osteogenic differentiation: let-7a and c, mir-22, 199a, 196a, 199b, mir-27, 98, 100, 615, 125b, and 195 (Figure 1A) (60). Moreover, evidence suggests mitochondria (61), ribosomes (62), and proteasomes (63) might also be enclosed and transferred by EVs to target cells. Knowledge of the biological cargo carried by EVs is continually developing, the development of integrated proteome, transcriptome, and lipidome databases—such as Vesiclepedia, EVpedia, and Exocarta (http://www.microvesicles.org/; http://evpedia.info; http://www.exocarta.org) now provide current data on EVs from a variety of sources.

EVs are a heterogeneous population of vesicles, thought to contain two predominant subtypes—exosomes (40–150 nm) and microvesicles (400–2,000 nm) (17). Exosomes are vesicles of endosomal origin formed by invagination of the endosomal membrane inside of multi-vesicular bodies (MVBs) with subsequent release of exosomes as a result of fusion of the MVBs with the plasma membrane. Microvesicles are released by budding directly from the plasma membrane (17). EVs biogenesis and purification strategies have been described in several published reviews (64).

As both subpopulations of EVs overlap in both size and density, separation of the populations within a biological sample is difficult. Therefore, EVs is used as collective descriptive term to describe the population of small vesicles released by most types of cells including MSCs (17).

In addition to carrying nucleic acids, MSCs derived EVs also reflect the protein characteristics of MSCs and contain proteins, associated with the therapeutic effects of MSCs: surface receptors (PDGFRB, EGFR, and PLAUR); signaling molecules (RRAS/NRAS, MAPK1, GNA13/GNG12, CDC42, and VAV2); cell adhesion molecules (CD29, CD44, CD73, FN1, EZR, IQGAP1, CD47, integrins, and LGALS1/LGALS3); and MSCs-associated antigens (CD9, CD63, CD81, CD109, CD151, CD248, and CD276) (Figure 1A) (63, 65). Recent transcriptomic and proteomic analysis of porcine MSCs derived EVs revealed that EVs were enriched in mRNAs encoding transcription factors and in proteins that support extracellular matrix remodeling, blood coagulation, inflammation, and angiogenesis (66).

Evidence also demonstrates that the biological activity of EVs is also similar to that of parental MSCs. EVs possess angiogenic, anti-apoptotic, and immunomodulatory properties similar to parental MSCs (67). It was shown that mouse MSCs derived EVs are enriched with VEGF protein and miR-210-3p via which they stimulate angiogenesis in ischemic limbs (68). In a rat model of spinal cord injury, administration of MSCs derived EVs resulted in a decrease in cellular apoptosis and inflammation at the injured site. This was accompanied by a decrease in the expression levels of proinflammatory cytokines (TNF-α and IL-1β) and an increase in anti-inflammatory cytokines IL-10 (69). In a model of traumatic acute lung injury (ALI) an increase of the survival rate and suppression of inflammatory response in experimental animals was also demonstrated (70). BM-MSCs derived exosomes reduced infarct size and improved cardiac function in rats after the acute myocardial infarction (71).

These results demonstrate the potential promise of MSCs derived EVs as an alternative to whole cell therapy. EVs efficiently mimic therapeutic effects of MSCs, whilst negating some of the safety concerns related to the use of MSCs, such as tumorigenicity and blood capillary embolism specific for MSCs based therapy (72). Currently, due to the lack of a nucleus, EVs do not fall into the category of advanced therapy medicinal products (ATMP) according to the European Medicines Agency (EMA) and the Food and Drug Administration (FDA) (73). Unlike gene therapy and cells-based approaches, EVs are not currently considered to be high-risk biologic drugs, but further conventional demonstrations of safety and efficacy in preclinical and clinical trials are essential (74).

## Immunomodulatory Activity of Extracellular Vesicles

MSCs derived EVs have shown immunosuppressive effects on many types of immune cells: dendritic cells, T cells, B cells and macrophages (75) (Figure 1B). It was shown that MSCs-derived exosomes and microvesicles exert similar immunosuppressive functions (72). However, it should be noticed that exosomes were more efficient in suppressing inflammation *in vivo* in inflammatory arthritis (76).

Reis et al. showed that EVs impaired antigen uptake by DCs, inhibited DCs maturation, secretion of pro-inflammatory cytokines IL-6 and IL-12p70 and increased their production of anti-inflammatory cytokine TGF-β (77). The immunomodulation was in part mediated by microRNAs (miR-21-5p, miR-142-3p, miR-223-3p, and miR-126-3p) which have known effects on DC maturation and were enriched in MSCs derived EVs (77) (Figure 1B).

Immunoregulation of T-cell mediated responses is an important tool to control autoimmune or inflammatory diseases. Treatment of T-cells with MSC derived EVs has been shown to result in a marked decrease in T-cell induced proliferation *in vitro* and downregulation of IFN-γ and TNF-α (78). Khare et al. also demonstrated the inhibitory effect of MSCs derived EVs on the proliferation of activated PBMCs and isolated T and B cells (47) (Figure 1B).

Studies conducted by Zhang et al., found that MSC derived EVs induced an M2-like phenotype in monocytes, induced T-cells to differentiate into regulatory T-cells and attenuated immune activity *in vivo* enhancing the survival of allogenic skin grafts in mice (79) (Figure 1B). Morrison et al. demonstrated that MSC derived EVs promote an anti-inflammatory and highly phagocytic macrophage phenotype through mitochondrial delivery (80). Taken together, these studies suggest that MSCs derived EVs retain the biological activity of the parental cells and are promising immunosuppressive instrument.

A number of different animal models have been used to study the immunomodulating activity of MSCs derived EVs (Table 1), demonstrating immunological activity and modification of the expression of both anti-inflammatory and pro-inflammatory cytokines. The immunomodulatory effect of EVs derived from human MSCs within animal model of pathogen/antigen induced tissue injury demonstrated a range of effects, including increases in survival in induced lung injury (81), decreased synovial lymphocyte counts and lowered TNF-α mRNA expression in synovitis joints (48), and increased regulatory T cells following concanavalin A induced liver injury (82). Additional preclinical studies in autoimmune uveoretinitis have shown MSCs derived EVs reduce the infiltration of inflammatory T cells in the eyes reducing the intensity of symptoms (83). Moreover, Shigemoto-Kuroda et al. demonstrated that MSC derived EVs suppressed Th1 and Th17 development, inhibited activation of APCs and T cells, increased expression of the immunosuppressive cytokine IL-10 and prevented development of uveoretinitis (84). Table 1 summarizes some more recent findings in the field of preclinical studies of EVs-MSCs based therapy of immune-mediated disorders. As seen from Table 1 EVs demonstrate the immunosuppression activity regardless of MSCs source. EVs derived from adipose-derived -MSCs, amnion-derived MSCs, and umbilical cord-MSCs are as effective as BM-MSCs (Table 1).

**Table 1:** Application of extracellular vesicles of mesenchymal stem cells for the therapy of immune-mediated diseases.

**Source of EVs**	**Model of disease**	**Observed effect**	**References**
**Inflammation associated diseases**			
Rat BM-MSCs	Cardiac ischemia	Inhibition of proliferation of T-cells *in vitro*, reduction of infarct size.	(85)
Pig AD-MSCs	Metabolic syndrome and renal artery stenosis	Attenuation of renal inflammation and fibrosis, improving of medullary oxygenation, renal blood flow, and glomerular filtration rate.	(86)
Human BM-MSCs	Local ischemic stroke (rat model)	Immune suppression 1 week after the injury, regeneration of blood vessels and nervous tissues.	(87)
Human BM-MSCs	Preterm brain injury (rat model)	Amelioration of inflammation, neuronal degeneration, reduction of microgliosis and prevention of astrogliosis.	(88)
Human BM-MSCs	Acute spinal cord injury	Attenuation of microglia activation, improving of locomotor recovery and mechanical sensitivity.	(89)
Mouse BM-MSC	Hepatic ischemia-reperfusion injury	Reduction of tissue necrosis, cells apoptosis, serum aminotransferase levels, expression of inflammatory cytokines (IL-6).	(90)
Rat amnion-derived MSCs	Liver fibrosis	Decrease of expression of inflammatory cytokines (TNF-α, Il-1β, Il-6, TGF-β), decrease of fiber accumulation, activation of Kupffer cells, and hepatic stellate cell.	(85)
**Autoimmune diseases**			
Human AD-MSCs	Multiple sclerosis (murine encephalomyelitis virus induced demyelinization)	Immunomodulation, decrease of inflammatory infiltrates, reducing of brain atrophy, increase of cell proliferation in the subventricular zone.	(89)
Mouse AD-MSCs	Autoimmune encephalomyelitis	Reduction of the severity of EAE by inhibiting of T cells extravasation in the inflamed central nervous system after the preventive administration of EVs.	(91)
Human BM-MSCs	Type 1 diabetes (mouse model)	Delay of the onset of T1D in mice, inhibition of activation of antigen-presenting cells and suppression of development of T helper 1 (Th1) and Th17 cells.	(84)
Mouse BM-MSCs	Rheumatoid arthritis	Inhibition of T lymphocyte proliferation, decrease of inflammation.	(76)
Human AD-MSCs	Atopic dermatitis (mouse model)	Reduction of symptoms, the levels of serum IgE, the number of eosinophils, infiltration of mast cells, CD86+, and CD206+ cells in skin lesions. Reduction of expression of inflammatory cytokines (IL-4, IL-23, IL-31 and TNF-α).	(92)
**Transplant rejection**			
Human Umbilical Cord-MSCs	aGVHD (mouse model)	Decrease of the symptoms, reduction of the mortality of the recipient mice, number of CD8+ T cells, reduction of serum levels of IL-2, TNF-α, IFN-γ and increase of the level of IL-10.	(93)
Human BM-MSCs	aGVHD (mouse model)	Prolongation of the survival of mice with aGVHD and reduction of the pathologic damage in organs, suppression of CD4+ and CD8+ T cells, suppression of the functional differentiation of T cells from a naive to an effector phenotype.	(94)

*BM-MSCs, bone marrow derived MSCs; AD-MSCs, adipose derived MSCs; TNF-α, tumor necrosis factor alpha; TGF-β, Transforming growth factor beta; IFN-γ, Interferon gamma; Th1, T helper cells; Th17, T helper 17 pro-inflammatory cells; EAE, encephalomyelitis; T1D, type 1 diabetes; aGVHD, acute graft-vs.-host disease*.

EVs circulate in body fluids disseminating throughout the body. To direct the immunosuppressive action of EVs, targeting strategies are actively being developed. Shamili et al. conjugated a myelin specific aptamer to the exosome surface and showed differences in the suppression of inflammatory response, demyelination process and severity of multiple sclerosis (95).

Clinical case studies to investigate the therapeutic potential of MSCs derived EVs have been conducted in patients with steroid-refractory acute graft-vs.-host disease (acute GvHD) (96). Treatment with EVs was reported to significantly improve GvHD symptoms in a 22-years female patient with severe cutaneous and intestinal GvHD, accompanied by a decrease in the level of IL-1b, TNF-a, IFN-γ secreted by patient-derived PBMCs (96).

Phase II/III clinical study on 20 patients with chronic kidney disease (CKD) showed that treatment with MSCs derived EVs improved the glomerular filtration rate (eGFR), serum creatinine level, blood urea and urinary albumin creatinine ratio (UACR) (97).

Despite these promising clinical studies, there are still limitations that need to be overcome in order to develop EVs-based medicines: (1) establishment of a recommended isolation protocol for large-scale preparation, purification and storage of EVs; (2) standardized protocols of EVs quantification, molecular, and physical EV characterization; and (3) defined quality control (QC) criteria for clinical use (74).

## Approaches for Increasing Plasma Membrane-Derived Vesicles Yield

Limited yield and labor intensive procedures for EV isolation has made large-scale production by pharmaceutical companies problematic and restrains the wider use of EVs in preclinical and clinical applications. The amount of the MSCs derived EVs isolated in the first clinical case study from supernatant of 4 × 10^7^ MSCs was 1.3–3.5 × 10^10^ particles or 0.5–1.6 mg (96). This dosage of EVs was defined as 1 unit and the patient received 4 units in total (96). To increase the yield of vesicles from MSCs, Mendt et al. suggested the use of bioreactor cultures of BM-MSCs (98). The authors were able to get 9.8–15.6 × 10^12^ exosomes per bioreactor run (98), a concentration sufficient for the treatment of one patient using the original clinical case protocol (97).

Further investigations of the biochemical and biophysical properties of membrane proteins and membrane organization are leading to novel approaches and continued improvements in current approaches for large scale vesicle production. Del Piccolo et al. induced the release of vesicles from CHO cells using “vesiculation buffer” (23). Cells were rinsed with a hypotonic buffer which induced cell swelling, then incubated the cells in a hypertonic solution. This osmotic buffer did not rupture the cells but stressed them sufficiently to increase the release of vesicles into solution (23). However, it remains to be determined how the increase in vesicle production alters the cargo and biological function of the resulting EVs.

Wu et al. produced vesicles from human retinal pigment epithelium (ARPE-19) cells by extruding the cell suspension through polycarbonate filter with 1 μm or 2 μm-pore size (20). The resulting population of vesicles consisted of two population—one with an average size 0.2 ± 0.1 μm and the smaller in number −0.8 ± 0.5 μm (20). A similar approach was used to produce vesicles from bone marrow derived MSCs via extrusion through a polycarbonate membrane with 3 μm pores. The authors were able to detect the functional active mitochondria inside the plasma membrane vesicles (21).

Plasma membrane-derived vesicles may be promising vector for the delivery of therapeutics. However, due to sporadic, unselective packing of cytoplasmic content into the plasma membrane-derived vesicles, these vesicles are not equal to the natural EVs, and the biological activity of such vesicles remains unknown and needs to be verified.

A more moderate approach to increase membrane vesicle production is treatment of cells with cytochalasin B (99). Cytochalasin B is a drug that causes disorganization of actin cytoskeleton (100). Treatment of cells with cytochalasin B resemble the disorganization of actin cytoskeleton by activated protease calpain during the natural process of microvesicle release (17, 101). Up to now EVs have been derived from HEK293 (99, 102, 103), 3T3 fibroblast (102), HUVECs (104), MDCKII-MDR1 (105), SH-SY5Y (22), and PC3 cells (106). Cytochalasin B-induced membrane vesicle (CIMVs) were used as vectors for nanoparticles and drugs delivery (102), decreasing the toxicity of chemotherapy *in vivo* by encapsulating doxorubicin (104). Cytochalasin B application to human cells promotes an increase in membrane vesicles yield by >100 fold (17). It was calculated that 17 ± 6% of the cell membrane transformed to CIMVs (99). The CIMVs released have a diameter of 100–1,000 nm (96%) which is comparable with naturally occurring EVs (22). Our studies of CIMVs have shown that their uptake by target cells is via heterophilic interaction of CIMVs membrane receptors with the surface proteins of target cells, this has a greater impact on CIMVs entry into target cells (106). Moreover, the biological activity of CIMVs is sufficient to stimulate capillary tube formation *in vitro* and angiogenesis *in vivo* by delivering growth factors (22). The use of Cytochalasin B to induced membrane vesicles (CIMVs) has advantages over endogenous EV production, including easier procedure of isolation, increased yield, more homogeneous composition due to the unselective mechanism of cytoplasmic content enclosing. The advantages of CIMVs production together with the evidence of retention of biological activity (22) strongly indicates that CIMVs may represent the next step toward the clinical use of EVs as therapeutic tools. However, the full immunomodulating properties of MSCs derived CIMVs remains to be fully evaluated.

## Conclusion

MSCs derived EVs retain the biological activity of parental MSCs and demonstrate a similar therapeutic potential. EVs stimulate the viability and proliferation of target cells and modulate the immune microenvironment. Therefore, the EVs of MSCs are potential therapeutic tools, which have advantages over cell therapy in terms of safety, ease of storage/transportation and clinical use. However, one of the major limitations of clinical adaption of EVs is the poor scalability of production. However, improved understanding of the physical properties of EVs and the mechanism of their biogenesis is leading to improved approaches to increase yield and uniformity of their production. Taken together, these data suggest that MSC derived EVs are a promising therapeutic tool for the treatment of immune-mediated disorders, including autoimmune diseases.

## Author Contributions

MG wrote the manuscript and created the tables and the figure. VJ edited the manuscript. MG and AR conceived the idea and edited the manuscript and figure.

### Conflict of Interest

The authors declare that the research was conducted in the absence of any commercial or financial relationships that could be construed as a potential conflict of interest.

## References

[1] Wang M Yuan Q Xie L. Mesenchymal stem cell-based immunomodulation: properties and clinical application. Stem Cells Int. (2018) 2018:3057624. 10.1155/2018/3057624 30013600 PMC6022321

[2] Gao F Chiu SM Motan DA Zhang Z Chen L Ji HL. Mesenchymal stem cells and immunomodulation: current status and future prospects. Cell Death Dis. (2016) 7:e2062. 10.1038/cddis.2015.327 26794657 PMC4816164

[3] Contreras RA Figueroa FE Djouad F Luz-Crawford P. Mesenchymal stem cells regulate the innate and adaptive immune responses dampening arthritis progression. Stem Cells Int. (2016). 2016:3162743. 10.1155/2016/3162743 27847522 PMC5101398

[4] Fu Y Karbaat L Wu L Leijten J Both SK Karperien M. Trophic effects of mesenchymal stem cells in tissue regeneration. Tissue Eng Part B-Rev. (2017) 23:515–28. 10.1089/ten.teb.2016.0365 28490258

[5] Vagner T Spinelli C Minciacchi VR Balaj L Zandian M Conley A. Large extracellular vesicles carry most of the tumour DNA circulating in prostate cancer patient plasma. J Extracell Vesic. (2018) 7:1505403. 10.1080/20013078.2018.1505403 30108686 PMC6084494

[6] Bellingham SA Coleman BM Hill AF. Small RNA deep sequencing reveals a distinct miRNA signature released in exosomes from prion-infected neuronal cells. Nucleic Acids Res. (2012) 40:10937–49. 10.1093/nar/gks832 22965126 PMC3505968

[7] Pitt JM Kroemer G Zitvogel L. Extracellular vesicles: masters of intercellular communication and potential clinical interventions. J Clin Invest. (2016) 126:1139–43. 10.1172/JCI87316 27035805 PMC4811136

[8] Bruno S Grange C Deregibus MC Calogero RA Saviozzi S Collino F. Mesenchymal stem cell-derived microvesicles protect against acute tubular injury. J Am Soc Nephrol. (2009) 20:1053–67. 10.1681/ASN.2008070798 19389847 PMC2676194

[9] Lai RC Arslan F Lee MM Sze NS Choo A Chen TS. Exosome secreted by MSC reduces myocardial ischemia/reperfusion injury. Stem Cell Res. (2010) 4:214–22. 10.1016/j.scr.2009.12.003 20138817

[10] Arslan F Lai RC Smeets MB Akeroyd L Choo A Aguor EN. Mesenchymal stem cell-derived exosomes increase ATP levels, decrease oxidative stress and activate PI3K/Akt pathway to enhance myocardial viability and prevent adverse remodeling after myocardial ischemia/reperfusion injury. Stem Cell Res. (2013) 10:301–12. 10.1016/j.scr.2013.01.002 23399448

[11] Herrera MB Fonsato V Gatti S Deregibus MC Sordi A Cantarella D. Human liver stem cell-derived microvesicles accelerate hepatic regeneration in hepatectomized rats. J Cell Mol Med. (2010) 14:605–18. 10.1111/j.1582-4934.2009.00860.x 19650833 PMC3060338

[12] Xin H Li Y Cui Y Yang JJ Zhang ZG Chopp M. Systemic administration of exosomes released from mesenchymal stromal cells promote functional recovery and neurovascular plasticity after stroke in rats. J Cereb Blood Flow Metab. (2013) 33:1711–5. 10.1038/jcbfm.2013.152 23963371 PMC3824189

[13] Rosland GV Svendsen A Torsvik A Sobala E McCormack E Immervoll H. Long-term cultures of bone marrow-derived human mesenchymal stem cells frequently undergo spontaneous malignant transformation. Cancer Res. (2009) 69:5331–9. 10.1158/0008-5472.CAN-08-4630 19509230

[14] Kunter U Rong S Boor P Eitner F Muller-Newen G Djuric Z. Mesenchymal stem cells prevent progressive experimental renal failure but maldifferentiate into glomerular adipocytes. J Am Soc Nephrol. (2007) 18:1754–64. 10.1681/ASN.2007010044 17460140

[15] Breitbach M Bostani T Roell W Xia Y Dewald O Nygren JM. Potential risks of bone marrow cell transplantation into infarcted hearts. Blood. (2007) 110:1362–9. 10.1182/blood-2006-12-063412 17483296

[16] Tatsumi K Ohashi K Matsubara Y Kohori A Ohno T Kakidachi H. Tissue factor triggers procoagulation in transplanted mesenchymal stem cells leading to thromboembolism. Biochem Biophys Res Commun. (2013) 431:203–9. 10.1016/j.bbrc.2012.12.134 23313481

[17] Gomzikova MO Rizvanov AA Current trends in regenerative medicine: from cell to cell-free therapy. Bionanoscience. (2017) 7:240–5. 10.1007/s12668-016-0348-0

[18] Chulpanova DS Kitaeva KV James V Rizvanov AA Solovyeva VV. Therapeutic prospects of extracellular vesicles in cancer treatment. Front Immunol. (2018) 9:1534. 10.3389/fimmu.2018.01534 30018618 PMC6037714

[19] Galieva LR James V Mukhamedshina YO Rizvanov AA. Therapeutic potential of extracellular vesicles for the treatment of nerve disorders. Front Neurosci. (2019) 13:163. 10.3389/fnins.2019.00163 30890911 PMC6411850

[20] Wu H Oliver AE Ngassam VN Yee CK Parikh AN Yeh Y. Preparation, characterization, and surface immobilization of native vesicles obtained by mechanical extrusion of mammalian cells. Integr Biol. (2012) 4:685–92. 10.1039/c2ib20022h 22543681

[21] Xu LQ Lin MJ Li YP Li S Chen SJ Wei CJ Preparation of plasma membrane vesicles from bone marrow mesenchymal stem cells for potential cytoplasm replacement therapy. J Vis Exp. (2017) 123:e55741-7. 10.3791/55741 PMC5608001 28570530

[22] Gomzikova MO Zhuravleva MN Miftakhova RR Arkhipova SS Evtugin VG Khaiboullina SF. Cytochalasin B-induced membrane vesicles convey angiogenic activity of parental cells. Oncotarget. (2017) 8:70496–507. 10.18632/oncotarget.19723 29050297 PMC5642572

[23] Del Piccolo N Placone J He L Agudelo SC Hristova K. Production of plasma membrane vesicles with chloride salts and their utility as a cell membrane mimetic for biophysical characterization of membrane protein interactions. Anal Chem. (2012) 84:8650–5. 10.1021/ac301776j 22985263 PMC3501251

[24] Siegel G Schafer R Dazzi F. The immunosuppressive properties of mesenchymal stem cells. Transplantation. (2009) 87(9 Suppl.):S45–9. 10.1097/TP.0b013e3181a285b0 19424005

[25] Yoo KH Jang IK Lee MW Kim HE Yang MS Eom Y. Comparison of immunomodulatory properties of mesenchymal stem cells derived from adult human tissues. Cell Immunol. (2009) 259:150–6. 10.1016/j.cellimm.2009.06.010 19608159

[26] Najar M Raicevic G Boufker HI Kazan HF De Bruyn C Meuleman N. Mesenchymal stromal cells use PGE2 to modulate activation and proliferation of lymphocyte subsets: combined comparison of adipose tissue, Wharton's Jelly and bone marrow sources. Cell Immunol. (2010) 264:171–9. 10.1016/j.cellimm.2010.06.006 20619400

[27] Zhang Q Shi S Liu Y Uyanne J Shi Y Shi S. Mesenchymal stem cells derived from human gingiva are capable of immunomodulatory functions and ameliorate inflammation-related tissue destruction in experimental colitis. J Immunol. (2009) 183:7787–98. 10.4049/jimmunol.0902318 19923445 PMC2881945

[28] Sato K Ozaki K Oh I Meguro A Hatanaka K Nagai T. Nitric oxide plays a critical role in suppression of T-cell proliferation by mesenchymal stem cells. Blood. (2007) 109:228–34. 10.1182/blood-2006-02-002246 16985180

[29] Ryan JM Barry F Murphy JM Mahon BP Interferon-gamma does not break, but promotes the immunosuppressive capacity of adult human mesenchymal stem cells. Clin Exp Immunol. (2007) 149:353–63. 10.1111/j.1365-2249.2007.03422.x 17521318 PMC1941956

[30] Aggarwal S Pittenger MF. Human mesenchymal stem cells modulate allogeneic immune cell responses. Blood. (2005) 105:1815–22. 10.1182/blood-2004-04-1559 15494428

[31] Li C Fu Y Wang Y Kong Y Li M Ma D. Mesenchymal stromal cells ameliorate acute allergic rhinitis in rats. Cell Biochem Funct. (2017) 35:420–5. 10.1002/cbf.3291 28940415 PMC5698748

[32] Li Y Liu J Liao G Zhang J Chen Y Li L. Early intervention with mesenchymal stem cells prevents nephropathy in diabetic rats by ameliorating the inflammatory microenvironment. Int J Mol Med. (2018) 41:2629–39. 10.3892/ijmm.2018.3501 29484379 PMC5846648

[33] Abdelmawgoud H Saleh A. Anti-inflammatory and antioxidant effects of mesenchymal and hematopoietic stem cells in a rheumatoid arthritis rat model. Adv Clin Exp Med. (2018) 27:873–80. 10.17219/acem/73720 29905411

[34] Yan L Jiang B Niu Y Wang H Li E Yan Y. Intrathecal delivery of human ESC-derived mesenchymal stem cell spheres promotes recovery of a primate multiple sclerosis model. Cell Death Discov. (2018) 4:28. 10.1038/s41420-018-0091-0 30131877 PMC6102276

[35] Connick P Kolappan M Patani R Scott MA Crawley C He XL. The mesenchymal stem cells in multiple sclerosis (MSCIMS) trial protocol and baseline cohort characteristics: an open-label pre-test: post-test study with blinded outcome assessments. Trials. (2011) 12:62. 10.1186/1745-6215-12-62 21366911 PMC3059276

[36] Dulamea A. Mesenchymal stem cells in multiple sclerosis - translation to clinical trials. J Med Life. (2015) 8:24–7. 25914733 PMC4397514

[37] Liang J Zhang H Kong W Deng W Wang D Feng X. Safety analysis in patients with autoimmune disease receiving allogeneic mesenchymal stem cells infusion: a long-term retrospective study. Stem Cell Res Ther. (2018) 9:312. 10.1186/s13287-018-1053-4 30428931 PMC6236873

[38] Haddad R Saldanha-Araujo F. Mechanisms of T-cell immunosuppression by mesenchymal stromal cells, what do we know so far? Biomed Res Int. (2014) 2014:216806. 10.1155/2014/216806 25025040 PMC4082893

[39] Bang OY Kim EH. Mesenchymal stem cell-derived extracellular vesicle therapy for stroke: challenges and progress. Front Neurol. (2019) 10:211. 10.3389/fneur.2019.00211 30915025 PMC6422999

[40] Zhang J Huang X Wang H Liu X Zhang T Wang Y. The challenges and promises of allogeneic mesenchymal stem cells for use as a cell-based therapy. Stem Cell Res Ther. (2015) 6:234. 10.1186/s13287-015-0240-9 26620426 PMC4665863

[41] Duffield JS Park KM Hsiao LL Kelley VR Scadden DT Ichimura T. Restoration of tubular epithelial cells during repair of the postischemic kidney occurs independently of bone marrow-derived stem cells. J Clin Invest. (2005) 115:1743–55. 10.1172/JCI22593 16007251 PMC1159124

[42] Biancone L Bruno S Deregibus MC Tetta C Camussi G. Therapeutic potential of mesenchymal stem cell-derived microvesicles. Nephrol Dial Transplant. (2012) 27:3037–42. 10.1093/ndt/gfs168 22851627

[43] Takahashi M Li TS Suzuki R Kobayashi T Ito H Ikeda Y. Cytokines produced by bone marrow cells can contribute to functional improvement of the infarcted heart by protecting cardiomyocytes from ischemic injury. Am J Physiol Heart Circulat Physiol. (2006) 291:H886–93. 10.1152/ajpheart.00142.2006 16603697

[44] den Haan MC Grauss RW Smits AM Winter EM van Tuyn J Pijnappels DA. Cardiomyogenic differentiation-independent improvement of cardiac function by human cardiomyocyte progenitor cell injection in ischaemic mouse hearts. J Cell Mol Med. (2012) 16:1508–21. 10.1111/j.1582-4934.2011.01468.x 22003890 PMC3823219

[45] Hodgkinson CP Bareja A Gomez JA Dzau VJ. Emerging concepts in paracrine mechanisms in regenerative cardiovascular medicine and biology. Circulat Res. (2016) 118:95–107. 10.1161/CIRCRESAHA.115.305373 26837742 PMC4874329

[46] Keshtkar S Azarpira N Ghahremani MH. Mesenchymal stem cell-derived extracellular vesicles: novel frontiers in regenerative medicine. Stem Cell Res Ther. (2018) 9:63. 10.1186/s13287-018-0791-7 29523213 PMC5845209

[47] Khare D Or R Resnick I Barkatz C Almogi-Hazan O Avni B. Mesenchymal stromal cell-derived exosomes affect mRNA expression and function of B-lymphocytes. Front Immunol. (2018) 9:3053. 10.3389/fimmu.2018.03053 30622539 PMC6308164

[48] Casado JG Blazquez R Vela FJ Alvarez V Tarazona R Sanchez-Margallo FM. Mesenchymal stem cell-derived exosomes: immunomodulatory evaluation in an antigen-induced synovitis porcine model. Front Vet Sci. (2017) 4:39. 10.3389/fvets.2017.00039 28377922 PMC5359696

[49] Shen B Liu J Zhang F Wang Y Qin Y Zhou Z. CCR2 positive exosome released by mesenchymal stem cells suppresses macrophage functions and alleviates ischemia/reperfusion-induced renal injury. Stem Cells Int. (2016) 2016:1240301. 10.1155/2016/1240301 27843457 PMC5098097

[50] Song JY Kang HJ Hong JS Kim CJ Shim JY Lee CW. Umbilical cord-derived mesenchymal stem cell extracts reduce colitis in mice by re-polarizing intestinal macrophages. Sci Rep. (2017) 7:9412. 10.1038/s41598-017-09827-5 28842625 PMC5573412

[51] Spinosa M Lu G Su G Bontha SV Gehrau R Salmon MD Human mesenchymal stromal cell-derived extracellular vesicles attenuate aortic aneurysm formation and macrophage activation via microRNA-147. FASEB J. (2018) 32:fj201701138RR 10.1096/fj.201701138RR PMC6181641 29812968

[52] Willis GR Fernandez-Gonzalez A Anastas J Vitali SH Liu X Ericsson M. Mesenchymal stromal cell exosomes ameliorate experimental bronchopulmonary dysplasia and restore lung function through macrophage immunomodulation. Am J Respir Crit Care Med. (2018) 197:104–16. 10.1164/rccm.201705-0925OC 28853608 PMC5765387

[53] Zhao H Shang Q Pan Z Bai Y Li Z Zhang H. Exosomes from adipose-derived stem cells attenuate adipose inflammation and obesity through polarizing M2 macrophages and beiging in white adipose tissue. Diabetes. (2018) 67:235–47. 10.2337/db17-0356 29133512

[54] Di Trapani M Bassi G Midolo M Gatti A Kamga PT Cassaro A. Differential and transferable modulatory effects of mesenchymal stromal cell-derived extracellular vesicles on T, B and NK cell functions. Sci Rep. (2016) 6:24120. 10.1038/srep24120 27071676 PMC4829861

[55] Blazquez R Sanchez-Margallo FM de la Rosa O Dalemans W Alvarez V Tarazona R. Immunomodulatory potential of human adipose mesenchymal stem cells derived exosomes on *in vitro* stimulated T cells. Front Immunol. (2014) 5:556. 10.3389/fimmu.2014.00556 25414703 PMC4220146

[56] Wen D Peng Y Liu D Weizmann Y Mahato RI. Mesenchymal stem cell and derived exosome as small RNA carrier and Immunomodulator to improve islet transplantation. J Control Release. (2016) 238:166–75. 10.1016/j.jconrel.2016.07.044 27475298

[57] Del Fattore A Luciano R Pascucci L Goffredo BM Giorda E Scapaticci M. Immunoregulatory effects of mesenchymal stem cell-derived extracellular vesicles on T lymphocytes. Cell Transplant. (2015) 24:2615–27. 10.3727/096368915X687543 25695896

[58] Budoni M Fierabracci A Luciano R Petrini S Di Ciommo V Muraca M. The immunosuppressive effect of mesenchymal stromal cells on B lymphocytes is mediated by membrane vesicles. Cell Transplant. (2013) 22:369–79. 10.3727/096368911X582769b 23433427

[59] Thakur BK Zhang H Becker A Matei I Huang Y Costa-Silva B. Double-stranded DNA in exosomes: a novel biomarker in cancer detection. Cell Res. (2014) 24:766–9. 10.1038/cr.2014.44 24710597 PMC4042169

[60] Kaur S Abu-Shahba AG Paananen RO Hongisto H Hiidenmaa H Skottman H. Small non-coding RNA landscape of extracellular vesicles from human stem cells. Sci Rep. (2018) 8:15503. 10.1038/s41598-018-33899-6 30341351 PMC6195565

[61] Islam MN Das SR Emin MT Wei M Sun L Westphalen K. Mitochondrial transfer from bone-marrow-derived stromal cells to pulmonary alveoli protects against acute lung injury. Nat Med. (2012) 18:759–65. 10.1038/nm.2736 22504485 PMC3727429

[62] Court FA Hendriks WT MacGillavry HD Alvarez J van Minnen J. Schwann cell to axon transfer of ribosomes: toward a novel understanding of the role of glia in the nervous system. J Neurosci. (2008) 28:11024–9. 10.1523/JNEUROSCI.2429-08.2008 18945910 PMC6671360

[63] Yu B Zhang X Li X. Exosomes derived from mesenchymal stem cells. Int J Mol Sci. (2014) 15:4142–57. 10.3390/ijms15034142 24608926 PMC3975389

[64] Konoshenko MY Lekchnov EA Vlassov AV Laktionov PP. Isolation of extracellular vesicles, general methodologies and latest trends. Biomed Res Int. (2018) 2018:8545347. 10.1155/2018/8545347 29662902 PMC5831698

[65] Kim HS Choi DY Yun SJ Choi SM Kang JW Jung JW. Proteomic analysis of microvesicles derived from human mesenchymal stem cells. J Proteome Res. (2012) 11:839–49. 10.1021/pr200682z 22148876

[66] Eirin A Zhu XY Puranik AS Woollard JR Tang H Dasari S. Integrated transcriptomic and proteomic analysis of the molecular cargo of extracellular vesicles derived from porcine adipose tissue-derived mesenchymal stem cells. PLoS ONE. (2017) 12:e0174303. 10.1371/journal.pone.0174303 28333993 PMC5363917

[67] Maas SLN Breakefield XO Weaver AM. Extracellular vesicles: unique intercellular delivery vehicles. Trends Cell Biol. (2017) 27:172–88. 10.1016/j.tcb.2016.11.003 27979573 PMC5318253

[68] Gangadaran P Rajendran RL Lee HW Kalimuthu S Hong CM Jeong SY. Extracellular vesicles from mesenchymal stem cells activates VEGF receptors and accelerates recovery of hindlimb ischemia. J Control Release. (2017) 264:112–26. 10.1016/j.jconrel.2017.08.022 28837823

[69] Huang JH Yin XM Xu Y Xu CC Lin X Ye FB. Systemic administration of exosomes released from mesenchymal stromal cells attenuates apoptosis, inflammation, and promotes angiogenesis after spinal cord injury in rats. J Neurotrauma. (2017) 34:3388–96. 10.1089/neu.2017.5063 28665182

[70] Li QC Liang Y Su ZB. Prophylactic treatment with MSC-derived exosomes attenuates traumatic acute lung injury in rats. Am J Physiol Lung Cell Mol Physiol. (2019) 316:L1107–17. 10.1152/ajpcell.00219.2018 30892077

[71] Zhang CS Shao K Liu CW Li CJ Yu BT. Hypoxic preconditioning BMSCs-exosomes inhibit cardiomyocyte apoptosis after acute myocardial infarction by upregulating microRNA-24. Eur Rev Med Pharmacol Sci. (2019) 23:6691–9. Hypoxic preconditioning BMSCs-exosomes inhibit cardiomyocyte apoptosis after acute myocardial infarction by upregulating microRNA-2 31378912 10.26355/eurrev_201908_18560

[72] Seo Y Kim HS Hong IS. Stem cell-derived extracellular vesicles as immunomodulatory therapeutics. Stem Cells Int. (2019) 2019:5126156. 10.1155/2019/5126156 30936922 PMC6413386

[73] Giebel B Kordelas L Borger V. Clinical potential of mesenchymal stem/stromal cell-derived extracellular vesicles. Stem Cell Invest. (2017) 4:84. 10.21037/sci.2017.09.06 29167805 PMC5676188

[74] Lener T Gimona M Aigner L Borger V Buzas E Camussi G. Applying extracellular vesicles based therapeutics in clinical trials - an ISEV position paper. J Extracell Vesic. (2015) 4:30087. 10.3402/jev.v4.30087 26725829 PMC4698466

[75] Burrello J Monticone S Gai C Gomez Y Kholia S Camussi G. Stem cell-derived extracellular vesicles and immune-modulation. Front Cell Dev Biol. (2016) 4:83. 10.3389/fcell.2016.00083 27597941 PMC4992732

[76] Cosenza S Toupet K Maumus M Luz-Crawford P Blanc-Brude O Jorgensen C. Mesenchymal stem cells-derived exosomes are more immunosuppressive than microparticles in inflammatory arthritis. Theranostics. (2018) 8:1399–410. 10.7150/thno.21072 29507629 PMC5835945

[77] Reis M Mavin E Nicholson L Green K Dickinson AM Wang XN. Mesenchymal stromal cell-derived extracellular vesicles attenuate dendritic cell maturation and function. Front Immunol. (2018) 9:2538. 10.3389/fimmu.2018.02538 30473695 PMC6237916

[78] van den Akker F Vrijsen KR Deddens JC Buikema JW Mokry M van Laake LW. Suppression of T cells by mesenchymal and cardiac progenitor cells is partly mediated via extracellular vesicles. Heliyon. (2018) 4:e00642. 10.1016/j.heliyon.2018.e00642 30003150 PMC6040605

[79] Zhang B Yin Y Lai RC Tan SS Choo AB Lim SK. Mesenchymal stem cells secrete immunologically active exosomes. Stem Cells Dev. (2014) 23:1233–44. 10.1089/scd.2013.0479 24367916

[80] Morrison TJ Jackson MV Cunningham EK Kissenpfennig A McAuley DF O'Kane CM. Mesenchymal stromal cells modulate macrophages in clinically relevant lung injury models by extracellular vesicle mitochondrial transfer. Am J Respir Crit Care Med. (2017) 196:1275–86. 10.1164/rccm.201701-0170OC 28598224 PMC5694830

[81] Monsel A Zhu YG Gennai S Hao Q Hu S Rouby JJ. Therapeutic effects of human mesenchymal stem cell-derived microvesicles in severe pneumonia in mice. Am J Respir Crit Care Med. (2015) 192:324–36. 10.1164/rccm.201410-1765OC 26067592 PMC4584251

[82] Tamura R Uemoto S Tabata Y. Immunosuppressive effect of mesenchymal stem cell-derived exosomes on a concanavalin A-induced liver injury model. Inflamm Regen. (2016) 36:26. 10.1186/s41232-016-0030-5 29259699 PMC5725906

[83] Bai L Shao H Wang H Zhang Z Su C Dong L. Effects of mesenchymal stem cell-derived exosomes on experimental autoimmune uveitis. Sci Rep. (2017) 7:4323. 10.1038/s41598-017-04559-y 28659587 PMC5489510

[84] Shigemoto-Kuroda T Oh JY Kim DK Jeong HJ Park SY Lee HJ. MSC-derived extracellular vesicles attenuate immune responses in two autoimmune murine models: type 1 diabetes and uveoretinitis. Stem Cell Rep. (2017) 8:1214–25. 10.1016/j.stemcr.2017.04.008 28494937 PMC5425726

[85] Teng XM Chen L Chen WQ Yang JJ Yang ZY Shen ZY. Mesenchymal stem cell-derived exosomes improve the microenvironment of infarcted myocardium contributing to angiogenesis and anti-inflammation. Cell Physiol Biochem. (2015) 37:2415–24. 10.1159/000438594 26646808

[86] Eirin A Zhu XY Puranik AS Tang H McGurren KA van Wijnen AJ. Mesenchymal stem cell-derived extracellular vesicles attenuate kidney inflammation. Kidney Int. (2017) 92:114–24. 10.1016/j.kint.2016.12.023 28242034 PMC5483390

[87] Hu B Chen S Zou M He Z Shao S Liu B. Effect of extracellular vesicles on neural functional recovery and immunologic suppression after rat cerebral apoplexy. Cell Physiol Biochem. (2016) 40:155–62. 10.1159/000452533 27855369

[88] Drommelschmidt K Serdar M Bendix I Herz J Bertling F Prager S. Mesenchymal stem cell-derived extracellular vesicles ameliorate inflammation-induced preterm brain injury. Brain Behav Immun. (2017) 60:220–32. 10.1016/j.bbi.2016.11.011 27847282

[89] Ruppert KA Nguyen TT Prabhakara KS Toledano Furman NE Srivastava AK Harting MT. Human mesenchymal stromal cell-derived extracellular vesicles modify microglial response and improve clinical outcomes in experimental spinal cord injury. Sci Rep. (2018) 8:480. 10.1038/s41598-017-18867-w 29323194 PMC5764957

[90] Haga H Yan IK Borrelli DA Matsuda A Parasramka M Shukla N. Extracellular vesicles from bone marrow-derived mesenchymal stem cells protect against murine hepatic ischemia/reperfusion injury. Liver Transplant. (2017) 23:791–803. 10.1002/lt.24770 28407355 PMC5495137

[91] Farinazzo A Angiari S Turano E Bistaffa E Dusi S Ruggieri S. Nanovesicles from adipose-derived mesenchymal stem cells inhibit T lymphocyte trafficking and ameliorate chronic experimental autoimmune encephalomyelitis. Sci Rep. (2018) 8:7473. 10.1038/s41598-018-25676-2 29748664 PMC5945853

[92] Cho BS Kim JO Ha DH Yi YW. Exosomes derived from human adipose tissue-derived mesenchymal stem cells alleviate atopic dermatitis. Stem Cell Res Ther. (2018) 9:187. 10.1186/s13287-018-0939-5 29996938 PMC6042362

[93] Wang L Gu Z Zhao X Yang N Wang F Deng A. Extracellular vesicles released from human umbilical cord-derived mesenchymal stromal cells prevent life-threatening acute graft-versus-host disease in a mouse model of allogeneic hematopoietic stem cell transplantation. Stem Cells Dev. (2016) 25:1874–83. 10.1089/scd.2016.0107 27649744

[94] Fujii S Miura Y Fujishiro A Shindo T Shimazu Y Hirai H. Graft-versus-host disease amelioration by human bone marrow mesenchymal stromal/stem cell-derived extracellular vesicles is associated with peripheral preservation of naive T cell populations. Stem Cells. (2018) 36:434–45. 10.1002/stem.2759 29239062

[95] Hosseini Shamili F Alibolandi M Rafatpanah H Abnous K Mahmoudi M Kalantari M. Immunomodulatory properties of MSC-derived exosomes armed with high affinity aptamer toward mylein as a platform for reducing multiple sclerosis clinical score. J Control Release. (2019) 299:149–64. 10.1016/j.jconrel.2019.02.032 30807806

[96] Kordelas L Rebmann V Ludwig AK Radtke S Ruesing J Doeppner TR. MSC-derived exosomes: a novel tool to treat therapy-refractory graft-versus-host disease. Leukemia. (2014) 28:970–3. 10.1038/leu.2014.41 24445866

[97] Nassar W El-Ansary M Sabry D Mostafa MA Fayad T Kotb E. Umbilical cord mesenchymal stem cells derived extracellular vesicles can safely ameliorate the progression of chronic kidney diseases. Biomater Res. (2016) 20:21. 10.1186/s40824-016-0068-0 27499886 PMC4974791

[98] Mendt M Kamerkar S Sugimoto H McAndrews KM Wu CC Gagea M. Generation and testing of clinical-grade exosomes for pancreatic cancer. JCI Insight. (2018) 3:99263. 10.1172/jci.insight.99263 29669940 PMC5931131

[99] Pick H Schmid EL Tairi AP Ilegems E Hovius R Vogel H. Investigating cellular signaling reactions in single attoliter vesicles. J Am Chem Soc. (2005) 127:2908–12. 10.1021/ja044605x 15740126

[100] Painter RG Whisenand J Mcintosh AT. Effects of cytochalasin-b on actin and myosin association with particle binding-sites in mouse macrophages - implications with regard to the mechanism of action of the cytochalasins. J Cell Biol. (1981) 91:373–84. 10.1083/jcb.91.2.373 7198123 PMC2111974

[101] Piccin A Murphy WG Smith OP. Circulating microparticles: pathophysiology and clinical implications. Blood Rev. (2007) 21:157–71. 10.1016/j.blre.2006.09.001 17118501

[102] Mao Z Cartier R Hohl A Farinacci M Dorhoi A Nguyen TL. Cells as factories for humanized encapsulation. Nano Lett. (2011) 11:2152–6. 10.1021/nl200801n 21486057

[103] Lim JH Park J Oh EH Ko HJ Hong S Park TH. Nanovesicle-based bioelectronic nose for the diagnosis of lung cancer from human blood. Adv Healthc Mater. (2014) 3:360–6. 10.1002/adhm.201300174 23868879

[104] Peng LH Zhang YH Han LJ Zhang CZ Wu JH Wang XR. Cell membrane capsules for encapsulation of chemotherapeutic and cancer cell targeting *in vivo*. Acs Appl Mater Interfaces. (2015) 7:18628–37. 10.1021/acsami.5b05065 26262951

[105] Eyer K Herger M Kramer SD Dittrich PS. Cell-free microfluidic determination of P-glycoprotein interactions with substrates and inhibitors. Pharm Res. (2014) 31:3415–25. 10.1007/s11095-014-1431-2 24928366

[106] Gomzikova M Kletukhina S Kurbangaleeva S Rizvanov A. Evaluation of cytochalasin B-induced membrane vesicles fusion specificity with target cells. Biomed Res Int. (2018) 2018:7053623. 10.1155/2018/7053623 29850552 PMC5911325

