# Supplementary material for: Therapeutic Application of Mesenchymal Stem Cells Derived Extracellular Vesicles for Immunomodulation
Source: Front Immunol. 2019 Nov 15;10:2663. doi: 10.3389/fimmu.2019.02663 (PMC6889906; doi:10.3389/fimmu.2019.02663)
Supplement: Supplementary file 1 [file Table_1.DOCX]

**Therapeutic application of mesenchymal stem cells derived extracellular vesicles for immunomodulation**

**Supplementary Data**

Table 1. Clinical trials of mesenchymal stem cells based therapy of autoimmune disorders

| Disease | Source of MSCs | Phase | First Posted Date, Initiating Company/ Sponsor, Country |
| --- | --- | --- | --- |
| Chronic Autoimmune Urticaria | Auto AD-MSC | Phase 1 | 03.03.2017; Celal Bayar University, Turkey. |
| Multiple Sclerosis | Auto AD-MSC | Phase 1  Phase 2 | 01.2010; Regional University Hospital in Málaga, University Hospital Virgen Macarena, Spain. |
|  |  |  | 30.12.2014; American CryoStem Corporation, Cayman Islands. |
|  | AD-MSC, no specification | Phase 1  Phase 2 | 04.10.2018; Stem Cell Medicine Ltd., Israel. |
|  | Auto MSC | Phase 1  Phase 2 | 21.11.2012; Karolinska Institute, Stockholm, Sweden. |
|  |  |  | 16.05.2013; University of Genova, Italy. |
|  |  |  | 12.09.2014; Ottawa Hospital Research Institute, Canada. |
|  |  |  | 26.10.2010; Instituto de Salud Carlos III, Spain. |
|  |  |  | 23.12.2008; Cleveland Clinic Mellen Center, USA. |
|  |  |  | 25.05.2012; Imperial College London, United Kingdom. |
|  | UC-MSC | Phase 1  Phase 2 | 25.07.2017; Jordan University Hospital Amman, Jordan. |
|  |  |  | 27.10.2015; Novo Cellular Medicine Institute LLP, India. |
|  |  |  | 16.04.2015; Genesis Limited, India. |
|  | Auto BM-MSC | Phase 1  Phase 2 | 31.03.2015; University Hospital, Toulouse, France. |
|  |  |  | 18.12.2018; Karolinska Institute, Sweden. |
|  |  |  | 21.06.2011; Royan Institute, Iran. |
|  |  |  | 14.01.2014; Germans Trias i Pujol Hospital Badalona, Spain. |
|  |  |  | 13.07.2015; Vall d'Hebron Research Institute, Spain. |
|  |  |  | 10.12.2012; Andalusian Initiative for Advanced Therapies, Spain. |
|  |  |  | 29.10.2008; Hadassah Medical Organization, Israel. |
|  |  |  | 18.06.2014; Hadassah Medical Center, Israel. |
|  |  |  | 03.03.2017; Stem Cells Arabia, Jordan. |
|  |  |  | 10.07.2013; Jordan University Hospital Amman, Jordan. |
|  |  |  | 10.01.2019; Brainstorm-Cell Therapeutics, USA. |
|  |  |  | 02.11.2006; University of Cambridge, United Kingdom. |
| Crohn's Disease | allo BM-MSC | Phase 1  Phase 2 | 22.02.2012; University Hospital Liège, Belgium. |
|  | UC-MSC | Phase 1  Phase 2 | 04.12.2013; Kang Stem Biotech Co., Ltd., South Korea. |
| Rheumatoid Arthritis | Auto AD-MSC | Phase 1  Phase 2 | 02.10.2018; Hope Biosciences, USA. |
|  | Allo AD-MSC | Phase 1  Phase 2 | 13.08.2012; TiGenix S.A.U., Spain. |
|  | Auto BM-MSC | Phase 2  Phase 3 | 10.06.2013; Royan Institute, Iran. |
|  |  | Phase 1 | 20.06.2016; Mashhad University of Medical Sciences, Iran. |
|  |  |  | 14.06.2017; MetroHealth Medical Center, USA. |
|  |  |  | 01.03.2017; Stem Cells Arabia, Jordan. |
|  | UC-MSC | Phase 1  Phase 2 | 13.01.2014; Stem Cell Institute, Panama. |
|  |  |  | 15.11.2013; Translational Biosciences, Panama. |
|  |  |  | 07.03.2012; Alliancells Bioscience Corporation Limited, China. |
|  |  |  | 31.12.2015; Shenzhen Hornetcorn Bio-technology Company, LTD, China. |
|  |  |  | 04.02.2019; Baylx Inc., USA. |
|  |  |  | 07.08.2018; Kang Stem Biotech Co., Ltd., South Korea. |
| Systemic Lupus Erythematosus | UC-MSC in Plasma-Lyte A solution | Phase 2 | 17.12.2015; Medical University of South Carolina, USA. |
|  | MSCs from UC, BM, AD, and DP and other in Plasma-Lyte A solution. | Phase 1 | 31.05.2017; Medical University of South Carolina, USA. |
|  | allo BM-MSC | Phase 1  Phase 2 | 17.06.2008; Nanjing Medical University, China. |
|  | UC-MSC | Phase 1  Phase 2 | 05.12.2012; Nanjing Medical University, China. |
|  |  |  | 01.07.2018; Saint-Louis Hospital, France. |
| Lupus Nephritis | Allo BM-MSC | Phase 1  Phase 2 | 17.09.2018; University Hospital Río Hortega, Spain. |
|  |  |  | 02.06.2017; Corestem, Inc., South Korea. |
|  | Auto MSCs | Phase 1  Phase 2 | 16.04.2008; Organ Transplant Institute, China. |
|  | UC-MSCs | Phase 2 | 09.07.2018; Lingyun Sun, China. |
|  |  |  | 28.02.2012; CytoMed & Beike, China. |
| Type 1 Diabetis | Auto BM-MSCs | Phase 1  Phase 2 | 25.03.2011; University of Sao Paulo, Brazil. |
|  |  |  | 05.12.2017; Central Hospital, Nancy, France. |
|  | Auto BM-MSC | Phase 2  Phase 3 | 07.07.2010; Southwest Hospital, Third Military Medical University, China. |
|  | UC-MSCs | Phase 1  Phase 2 | 13.10.2010; Stem Cell Research Center of Medical School Hospital of Qingdao University,China. |
|  |  |  | 16.06.2011; Fuzhou General Hospital, China. |
|  |  |  | 2.06.2011; Shenzhen Beike Bio-Technology Co., Ltd., China. |
|  |  |  | 05.05.2016; The Affiliated Nanjing Drum Tower Hospital of Nanjing University Medical School, China. |
|  | Cotransplantation of allograft Islet and auto MSCs | Phase 1  Phase 2 | 28.03.2008; Fuzhou General Hospital, China. |
|  | Auto MSCs | Phase 2 | 07.02.2014; Uppsala University Hospital, Sweden. |
|  | Auto BM-MSC and allo UC-MSC combined with PRP | Phase 1  Phase 2 | 01.04.2017; Van Hanh General Hospital University of Science, Vietnam. |
|  | Allo BM-MSC | Phase 2 | 08.09.2016; Clínica Alemana de Santiago, Chile. |
|  | Allo AD-MSC with auto BM-MSC | Phase 1 | 20.10.2016; Sophia Al-Adwan, University of Jordan, Jordan. |
|  | Auto BM-MSC and UC-MSCs | Phase 1 | 14.06.2010; Cellonis Biotechnology Co. Ltd., China. |
|  | PROCHYMAL® - adult MSCs | Phase 2 | 04.06.2008; Mesoblast International, USA. |
|  | Men-MSCs | Phase 1  Phase 2 | 21.12.2011; Evans Biosciences Co., Ltd.,Zhejiang University, China. |
| Neuromyelitis Optica Spectrum Disorders | Auto MSCs | Phase 2 | 25.09.2014; Tianjin Medical University General Hospital, China. |
| Sjögren's Syndrome | Allo MSCs | Phase 1  Phase 2 | 06.08.2009; The Affiliated Nanjing Drum Tower Hospital of Nanjing University Medical School, China. |
| Hepatitis | UC-MSCs | Phase 1  Phase 2 | 10.08.2012; Beijing 302 Hospital, China. |

**UC** – umbilical cord; **BM** – bone marrow; **AD** – adipose derived; **DP** – dental pulp; **auto AD-MSCs** – autologous adipose derived mesenchymal stem cells; **allo AD-MSC** – allogenous adipose derived mesenchymal stem cells; **auto BM-MSCs** - autologous bone-marrow derived mesenchymal stem cells; **allo BM-MSCs** - allogenous bone-marrow derived mesenchymal stem cells; **UC-MSCs** - umbilical cord derived mesenchymal stem cells; **allo UC-MSC** - allogenous umbilical cord derived mesenchymal stem cells; **PRP** - platelet-rich plasma.
